# Population Receptive Field Dynamics in Human Visual Cortex

**DOI:** 10.1371/journal.pone.0037686

**Published:** 2012-05-23

**Authors:** Koen V. Haak, Frans W. Cornelissen, Antony B. Morland

**Affiliations:** 1 Laboratory for Experimental Ophthalmology and BCN Neuroimaging Center, University Medical Center Groningen, University of Groningen, Groningen, The Netherlands; 2 York Neuroimaging Centre, Department of Psychology, University of York, York, York, United Kingdom; 3 Centre for Neuroscience, Hull-York Medical School, York, United Kingdom; University of Regensburg, Germany

## Abstract

Seminal work in the early nineties revealed that the visual receptive field of neurons in cat primary visual cortex can change in location and size when artificial scotomas are applied. Recent work now suggests that these single neuron receptive field dynamics also pertain to the neuronal population receptive field (pRF) that can be measured in humans with functional magnetic resonance imaging (fMRI). To examine this further, we estimated the pRF in twelve healthy participants while masking the central portion of the visual field. We found that the pRF changes in location and size for two differently sized artificial scotomas, and that these pRF dynamics are most likely due to a combination of the neuronal receptive field position and size scatter as well as modulatory feedback signals from extrastriate visual areas.

## Introduction

Visual neurons respond to a limited part of the visual field. This portion of the visual field is known as the receptive field. To infer the distribution of receptive field location and size across human visual cortex, functional magnetic resonance imaging (fMRI) can be used [1]–[6]. Due to limited spatial resolution, however, fMRI can only capture the central tendency of many neuronal receptive fields. Hence, the region of visual space that stimulates a voxel is referred to as the population receptive field (pRF) [7].

**Figure 1:**
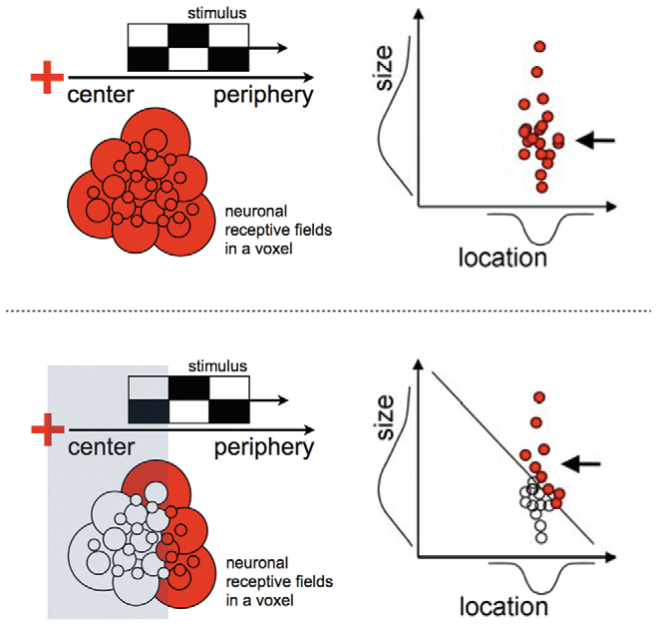
How changed population receptive fields may emerge from partially stimulating the visual field. See also [8], [14]. If a stimulus (the checkered block) moves over a region of visual space that covers all neurons' receptive fields (top row), all neurons should respond and contribute to the pRF estimate (as indicated by red shading). If, in contrast, a stimulus moves over a more restricted region of visual space that covers a more limited number of neurons', only a subset of neurons will respond and contribute to the pRF estimate. This is true in the masked conditions we used (bottom row). Therefore, the pRF estimates can change as a result of a stimulus change even when the underlying neuronal receptive field properties remain constant. When a central mask is applied, it is also true that neuronal receptive fields contributing to the pRF estimate have, on average, more eccentric locations in the visual field than those that were silenced by masking the stimulus. This is why more eccentric pRF estimates emerge. Finally, as illustrated by the pair of plots on the right, larger pRF estimates (indicated by the arrowheads) also emerge because the active neurons (red circles) during the masked conditions have receptive field that are more likely to be larger. The diagonal line in the lower right plot corresponds to the sum of the receptive field size and location equating to the size of the mask.

In previous work [8], we studied the mean pRF in the cortical lesion projection zone of patients with macular degeneration (MD). Compared with age-matched controls, we found that in MD patients the mean pRF was larger and also corresponded to a more peripheral location in the visual field. This result would have been taken as evidence for cortical reorganization, if it were not that the same changes occurred when the effect of an artificial scotoma was examined in a group of healthy participants. Rather, it seemed that central pRFs can be displaced and enlarged simply by silencing central visual field stimulation (Figure 1).

In the present study, we further examined these pRF dynamics for two differently sized artificial scotomas, asking whether the effect can be traced down to the level of single pRFs and what neuronal mechanisms could be causing it. For two differently sized artificial scotomas we found that some voxels in retinotopic representations of the center of the visual field also responded when more peripheral locations were stimulated alone. The effects we document are most likely due to a combination of the neuronal receptive field position and size scatter as well as modulatory feedback signals from extrastriate visual cortex.

## Methods

### Subjects

We report on measurements from twelve subjects (ages 18–41) with normal or corrected-to-normal vision. All subjects gave informed written consent according to procedures that followed the tenets of the Declaration of Helsinki and were approved by the York Neuroimaging Research Governance committee.

### Stimuli

**Figure 2:**
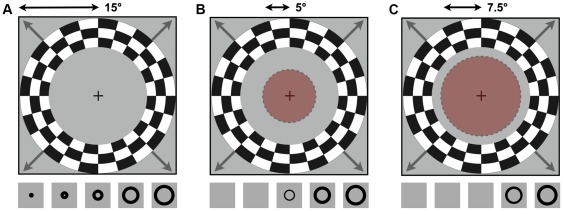
Illustration of the expanding ring stimuli in each experimental condition. (**a**) Stimulus schematic of the full-field condition. The maximum stimulus radius was 15°. The bottom panels show how the stimulus changes over time. (**b**, **c**) Stimulus schematic of the 5° and 7.5° masked conditions, respectively (masks are shown in opaque red). Bottom panels indicate the resulting stimulus sequence. For clarity, only 5 of the 12 ring positions are shown.

The visual stimuli (Figure 2) consisted of expanding ring apertures in a mean luminance gray background that exposed a high contrast (100%) flickering radial checkerboard pattern. The expanding ring aperture comprised three rings of the checkerboard pattern that increased in angular extent to a maximum of 15°. A new ring at the center replaced each ring as it approached the outer border of the stimulated region of the visual field. During all experimental conditions, the expanding ring stimuli had a period of 36 s and were repeated for seven full cycles. In separate scans, the subjects were either shown the full stimulus or masked versions. The masks consisted of a centrally placed static disk at mean luminance gray so that the central portion of the visual field was constant throughout the scan. Two masks were used such that the constant portion of the visual field subtended either a 5.0° or 7.5° radius. During all experimental conditions, participants were asked to fixate a red fixation cross that was placed at the center of the screen. This fixation cross, which was visible throughout each scan, ensured that participants maintained fixation in each condition. The visual stimuli were generated with Matlab (Mathworks Inc.) and controlled by MatVis (Neurometrics Institute). The stimuli were presented using an ImagePro 8942 LCD projector and rear projected onto a translucent acrylic screen situated in the bore of the MRI scanner, behind the subject's head. Subjects viewed the stimuli via a mirror mounted on the head coil.

### Magnetic resonance imaging

Functional and structural MRI data were acquired using an 8-channel, phase-array head coil on a GE 3-Tesla Signa HD Excite scanner. For structural data, multi average, whole-head T1 weighted anatomical volumes (1×1×1.13 mm^3^) were acquired for each subject. For functional data, gradient-echo pulse sequences were used to measure the T2* BOLD signal (TR/TE = 3000/30 ms, FOV = 28.8 cm, 128×128 matrix, 25 contiguous slices with 3 mm slice thickness). Images were read out using an EPI sequence. Magnetization was allowed to reach a steady state by discarding the first five volumes. For each of six scans (two for each condition), these first five volumes were followed by the acquisition of a further 84 volumes.

### Data preprocessing

Data were analyzed using the mrVISTA toolbox (http://white.stanford.edu/software) and FSL (http://www.frmib.ox.ac.uk/fsl). For anatomical data, the occipital cortices in the acquired anatomical volumes were manually segmented into white and gray volumes. For functional data, the images were corrected for spatial inhomogeneity. Motion correction was performed and functional time series were high-pass filtered to remove baseline drifts, after which they were converted to percent signal change (i.e., Δ% = 100•[*x*/mean(*x*)−1]).

### Regions-of-interest (ROI) definition

**Figure 3:**
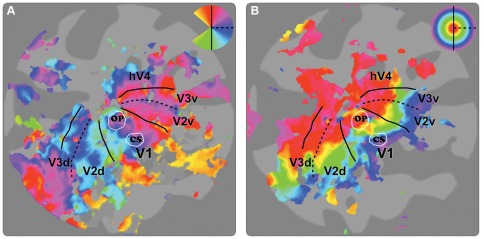
ROI locations on a flattened cortical surface of one of the participants. (**a**) The polar angle maps are indicative to the locations of the early visual areas. (**b**) The eccentricity maps are indicative to the centrals and peripheral visual field representations. The occipital pole (OP) ROIs are located in V1 at the border between the calcarine sulcus and the occipital pole, a region that responds to stimuli presented to the center of the visual field. The calcarine sulcus (CS) ROI is also located in V1, but more anteriorly in the calcarine sulcus, which responds to more peripherally presented stimuli. Insets indicate the color maps that define the visual field representation. Solid black lines indicate the representation of the vertical meridian, and dashed black lines indicate the representation of the horizontal meridian. Solid white lines indicate the borders of the ROIs.

ROIs were defined using an algorithm that gathered all contiguous gray matter in a circular patch centered on a selected point in the high-resolution anatomical data. Based on anatomical criteria, two ROIs were chosen in each hemisphere of each subject: the OP ROI at the border between the calcarine sulcus and the occipital pole, a region in the primary visual cortex (V1) that represents activity from central retina, and the CS ROI located more anteriorly in the calcarine sulcus that represents V1 activity from more peripheral retina (Figure 3). Both ROIs were 20 mm diameter in all participants. To exclude the possibility that the OP ROIs included extrastriate cortex, V1 was defined in both hemispheres from separate scans that included (unmasked) rotating wedge-stimuli (note that the wedge data were not included in any other analyzes; see ref [8] for a description of the wedge-stimuli). All voxels that did not fall within V1 were excluded from the ROIs.

### Population receptive field modeling

Population receptive field (pRF) modeling was performed to assess the degree to which the time series of the voxels in the ROIs fitted a series of circular symmetric two-dimensional Gaussian receptive field models [6]. Crucially, all experimental conditions were analyzed based on model predictions that assumed full-field stimulation. That is, we did not constrain the potential pRFs to respond only to stimulated parts of the visual field. This is essential if we wish to compare voxel response properties under different conditions; we can change only one thing – the stimulus – while keeping the analysis procedure constant. As in previous work [6], [8], [9], best fitting models were retained if they accounted for more than 15% of the variance of the time series. Given that the time-series consisted of 84 time-frames, this threshold corresponded to a significance level of *p*<0.001 (uncorrected) [10]. This procedure was carried out for each condition separately. Importantly, we ensured that for the occipital pole ROIs our subsequent analyzes only included voxels for which the pRF center eccentricity was less than 5.0° and 7.5° when the full stimulus was presented, respectively.

### Estimating temporal phase and duty-cycle

To verify the pRF modeling approach, a one-dimensional variant of the two-dimensional pRF modeling method was also performed [4]. In this analysis the temporal phase of the time-series was computed for each individual voxel whose best-fitting 2D pRF model explained more than 15% of the time-series variance by finding the phase of their fundamental Fourier components. Furthermore, in the case of ring-stimuli, the spread of the circular symmetric Gaussian pRF model is proportional to the duty-cycle of the time-series. This duty-cycle was estimated by generating a number of time-series predictions from a set of square waves with 100 different phase delays (equally spaced between 0–2π) and 100 different duty-cycles (equally spaced between 0–100%). As in the pRF modeling approach, the time-series predictions were convolved with a two-gamma hemodynamic response function. The best-fitting duty-cycle was then found by minimizing the residual sum of squares between the fMRI and the predicted time-series.

### Statistical analyzes

Statistics were calculated using functions of the Matlab Statistical Toolbox. Taking into consideration the unequal number of points contributed by each subject, all reported ranges correspond to the 95% confidence intervals of the jackknifed (leave one subject out) mean. This procedure allowed us to capture the between-subject variance without completely disregarding the between voxel variance within subjects.

## Results

**Figure 4:**
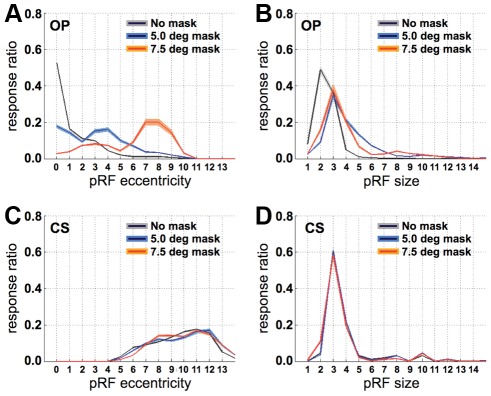
Response distributions for the unmasked and masked conditions. **A.** Response ratio (i.e., the number of responsive voxels per bin divided by the total number of responsive voxels) versus pRF eccentricity for the unmasked and masked conditions for the OP region-of interest. **B.** Response ratio versus pRF size for the unmasked and masked conditions in the OP region-of interest. **C.** Response ratio versus pRF location for the unmasked and masked conditions in the CS region-of interest. **D.** Response ratio versus pRF size for the unmasked and masked conditions in the CS region-of interest. Note that in the masked conditions the pRFs shift away from their original location in the OP regions (**A**) but not in the CS region (**C**). The pRFs in the OP regions are also larger for the masked conditions in the OP regions (**B**), but not in the CS region (**D**). Gray, blue and orange shadings indicate the jackknifed 95% confidence interval for the unmasked and the two masked conditions, respectively.

We first evaluated the response distributions of the pRF location and size for the unmasked and masked conditions (Figure 4). In the OP region, the full stimulus resulted in central pRFs that were small. In the CS region, the full stimulus led to peripheral pRFs that were large. When the stimulus was masked, however, the response distributions of pRF location and size in the OP region shifted towards greater eccentricities and larger sizes, respectively. These shifts and increases occurred for both masks, but not for the CS region. Of note is also the pRF size in the OP region for the full stimulus, which is rather large compared to some of the previous studies using the same pRF modeling method [5], [6]. This feature has also been observed by Winawer et al. [9] and may be due to B_0_ field distortions related to the presence of several dural sinuses, voxel size differences, or the fact that the ring apertures were large relative to the receptive field sizes in V1. In the following, we assume that these limitations apply equally to all experimental conditions.

In the OP region, far fewer voxels had a reliable pRF when the stimulus was masked compared to when it was not (no mask: 56%, 5° mask: 27%, 7.5° mask: 8%). Therefore, it is possible that the distribution shifts in Figure 4 resulted from sampling, in the masked conditions, only those voxels that had large and eccentric pRFs in the first place. To avoid such sampling bias, we restricted all further analyzes to voxels that responded above threshold (more than 15% variance explained) in both the unmasked and at least one of the two masked conditions. For these ‘matched’ voxels we found that there were significant shifts in pRF location of 1.45°±0.08° and 4.13°±0.2° for the 5.0° and 7.5° masks, respectively. Significant increases in pRF size were also found; 1.43°±0.05° and 0.92°±0.1° for the 5.0° and 7.5°masks, respectively. Similar results were also obtained using a higher threshold for the variance explained by the model. For all voxels that responded in both masked and unmasked conditions explaining more than 20% of time-series variance, the location changes were 1.44°±0.07° (5.0° mask) and 4.19°±0.2° (7.5° mask), and the size changes were 1.13°±0.03° (5.0° mask) and 0.39°±0.1° (7.5° mask). Therefore, our results do not appear to be crucially dependent on a specific statistical threshold.

**Figure 5:**
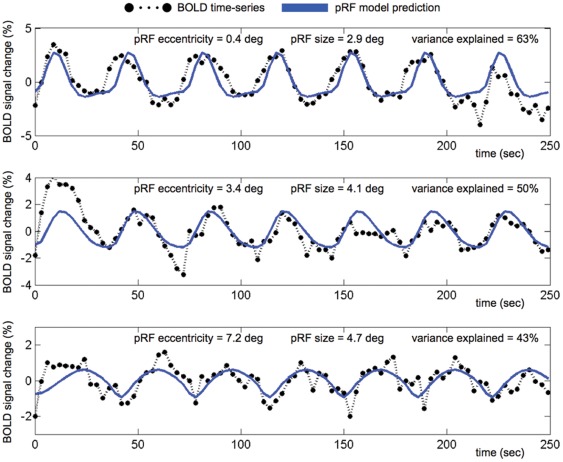
Examples of the model fits. Model fits to BOLD time-series are shown for a voxel in the OP region that explained more than 15% of the time-series variance in the unmasked (top) and both of the two masked conditions (middle and bottom). The BOLD time-series show increasingly broader peaks and more pronounced phase-shifts corresponding to an increased estimate of the pRF size and location, respectively. Note that the y-axes in the three panels have different scales.

Given the pRF changes observed, differences should be clearly visible in the individual time-series and their model fits. That is, a shift in pRF position should be visible as a phase-shift in the individual time-series, and an increased pRF size should be visible as broader peaks corresponding to longer activation. Figure 5 shows two examples of the time-series and model fits during the masked and unmasked conditions for the same voxel. The predicted time-series generated by the best-fitting models in this example explained 63% for the unmasked condition, and 50% and 43% for the masked conditions. As expected, compared with the time-series for the unmasked condition, the peaks in the masked conditions were shifted in time and corresponded to longer activation durations.

That the voxel time-series in Figure 5 exhibited a phase-shift and broader peaks when the stimulus was masked also suggests that the observed pRF changes did not emerge from the particulars of the pRF modeling approach. In the present experiment we basically fitted a two-dimensional pRF model to a one-dimensional data set (eccentricity only). It could therefore be argued that the pRF changes reflect unstable model fits due to over-fitting. Hence, we also assessed whether the pRF changes could be derived using a one-dimensional variant of the pRF modeling method that estimates the phase and duty-cycle of the voxel time-series [4]. Indeed, for all voxels that responded above threshold in both the unmasked condition and at least one of the two masked conditions, we found that the phase of the time-series shifted from 0.67 to 1.51 radians for the 5.0° mask, and from 1.08 to 2.82 radians for the 7.5° mask. Converted to eccentricity these values corresponded to 1.59°, 3.61°, 2.57° and 6.72°, respectively. With regard to verifying the observed pRF size change, the estimated duty-cycle also showed a substantial increase as a result of the two masks: for the 5° mask the duty-cycle increased from 42% to 45%, and for the 7.5° mask the duty-cycle increased from 44% to 49%. Of note is also that these one-dimensional duty-cycle estimates are very similar to those reported previously. For example, based on a similar ring-only stimulus prescription, Smith et al. [4] found that the duty-cycle in V1 ranged between 40% and 60% for eccentricities spanning the central 10 degrees of visual angle.

**Figure 6:**
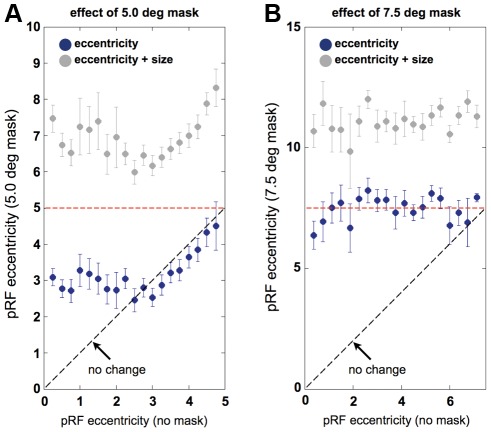
The effect of masking the central portion of the visual field on pRF eccentricity. **A.** The population receptive field (pRF) eccentricity within the OP ROI derived for the 5.0° masked condition as function of the same measure derived from the unmasked condition is plotted on blue. The sum of pRF location and size for the 5.0° masked condition are also plotted as a function of pRF location for the unmasked condition (gray). **B.** The same plot as in **A** but for the 7.5° masked condition. The dashed black lines indicate the predicted result if voxels that responded in the same way in both conditions. The dashed red lines in each plot show the borders of the masks. Error-bars indicate the standard error. Note that the axes in the two panels have different scales.

Figure 6 illustrates further the relationship between the pRF location estimates obtained from the occipital pole in the unmasked and both masked conditions. For most voxels the pRF locations fall above the black dotted line of unit slope, indicating a shift to more eccentric locations as a result of the mask. However, there are also voxels that exhibit pRF locations that lie well within the masked zone and it is not clear whether these voxels are genuinely driven by peripheral stimuli. To examine this, we tested the prediction that the sum of pRF location and size exceeded the radius of the masks. The gray dots in Figures 6 indicate that the vast majority of voxels (81% for the 5.0° mask and 94% for the 7.5° mask) responded to stimulus positions beyond the masked region, given each voxel's combination of the pRF location and size. Importantly then, the pRF estimates for the voxels in the occipital pole indicate that voxels are genuinely visually driven by eccentric stimuli. In the unmasked condition only 37% of the pRFs of the matched voxels extended beyond 5° and 32% beyond 7.5°, indicating again that it was the mask that caused the peripheral responses in the occipital pole regions.

There are two relationships between pRF size and location that should emerge if the pRF parameters are capturing information about neural responses. The first simply reflects what has already been demonstrated in previous pRF analyzes and is well known from electrophysiological measurements, namely, that in the unmasked condition there should be a positive relationship between the receptive field size and location. The second captures the feature that if a region of cortex normally encodes a small eccentricity, it would require a large receptive field to be driven by an eccentric stimulus and vice versa. This should lead to a negative relationship between receptive field size and location in the occipital pole region of interest under masked conditions. Indeed, for both the 5° mask and 7.5° the correlation coefficient between the pRF eccentricity and size changed from positive (*r* = 0.248±0.01; *r* = 0.421±0.02) to negative (*r* = −0.006±0.02; *r* = −0.416±0.02).

**Figure 7:**
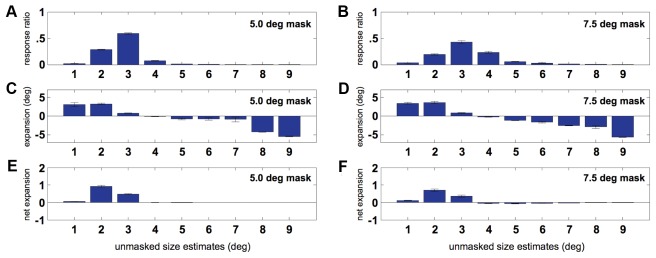
Response and expansion distributions across the different pRF sizes. **A.** Response distribution of pRF sizes estimated for the unmasked conditions for voxels that responded in the 5.0° masked condition as well as the full-field condition. **B.** Response distribution of pRF sizes estimated for the unmasked conditions for voxels that responded in the 7.5° masked condition as well as the full-field condition. **C.** Mean pRF expansion for voxels that responded in the 5.0° masked condition as well as the full-field condition. **D.** Mean pRF expansion for voxels that responded in the 7.5° masked condition as well as the full-field condition. **E.** The net effect of the change in pRF size, as measured by the product of the mean change and the number of voxels per bin induced by the 5.0° mask. **F.** The net effect of the change in pRF size, as measured by the product of the mean change and the number of voxels per bin induced by the 7.5° mask. The error-bars for each bin are jackknife estimates of the 95% confidence interval.

While we report an overall increase in pRF size in masked conditions, our data also revealed decreases in size for some voxels. In order to establish the source of such decreases, we examined the change in sizes of pRFs for all voxels binned by the receptive field size estimates for the unmasked condition (Figure 7). First, the pRF size frequency (response ratio) distributions are given for the unmasked condition (Figures 7A and 7B). The bar graphs in Figures 7C and 7D clearly show that increases in pRF size are observed for voxels that recorded small (under 4°) receptive field sizes in the unmasked condition. However, the voxels that had large pRFs (over 4°) in the unmasked condition exhibited decreases in pRF size. It is also clear that large (over 4°) pRFs are rather uncommon (Figures 7A and 7B). Because the number of voxels with large pRFs is small, the net change (frequency×change) is an expansion (Figures 7E and 7F).

**Figure 8:**
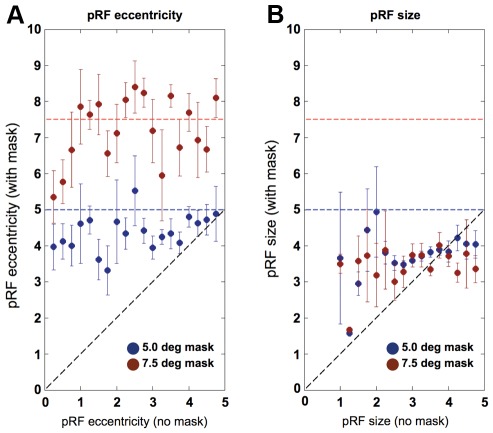
Effect of masking the central visual field for voxels that responded in all three conditions. **A.** The pRF eccentricity within the OP ROI derived for the 5.0° (blue) and 7.5° (red) masked conditions as function of the same measure derived from the unmasked condition. **B.** The pRF size within the OP ROI derived for the 5.0° (blue) and 7.5° (red) masked conditions as function of the pRF size derived from the unmasked condition. The dashed black lines indicate the predicted result if voxels that responded in the same way in both conditions. The dashed blue and red lines represent the borders of the 5.0° and 7.5° masks, respectively. Error-bars indicate the standard error.

The results presented thus far concerned voxels that responded above threshold during the unmasked condition and one of the two masked conditions, but not necessarily during all three conditions. This was done because the fraction of voxels responding during all three conditions is much smaller than the fraction of voxels responding in the unmasked and one of the two masked conditions. In addition, it should be noted that there is a genuine effect if the pRF eccentricity moved from 5.5 to 7 degrees of visual angle for the condition with the 7.5° mask, but not for the 5° mask, and excluding all voxels beyond the extent of the smaller mask would bias the results for the condition with the larger mask. However, with this in mind, to analyze directly the relationship between two mask sizes, we nevertheless compared the effect of applying an increasingly larger mask (from 0° to 5° to 7.5°) in all voxels that responded above threshold during all three conditions for which the pRF eccentricity in the unmasked condition did not exceed 5°. Figure 8A shows that the pRFs for these voxels shifted away lawfully from their original location towards the fringe of mask. Figure 8B further shows that the pRF size increases to roughly 3.5° in the masked conditions (see also Figure 4B).

**Figure 9:**
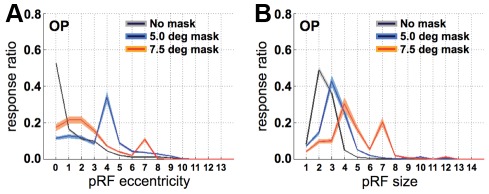
The effect of modeling the presence of a central mask. **A.** Response ratio (i.e, the number of responsive voxels per bin divided by the total number of responsive voxels) versus pRF eccentricity for the unmasked and masked conditions for the OP region-of interest. **B.** Response ratio versus pRF size for the unmasked and masked conditions in the OP region-of interest. Gray, blue and orange shadings indicate the jackknifed 95% confidence interval for the unmasked and the two masked conditions, respectively.

Finally, as indicated in the methods section, the above results were obtained from model predictions that assumed full-field stimulation. This was done so to avoid changing both the stimulus and the modeling procedure. It is, however, possible that this choice made the fitting procedure more unstable during the masked conditions. We therefore repeated the model fitting procedure for the masked conditions with the mask included to generate the model predictions. Being conscious of the fact that any pRF changes found could now be due to changing the modeling rather than the stimulus, it should still be possible to detect the above-mentioned pRF changes. Indeed, Figure 9 shows that the pRFs in the OP ROIs shift towards ∼4° when the 5° mask was applied, and to ∼7° when masking the central 7.5° of the visual field. Furthermore, the pRF size in the OP ROI increased with ∼1° and ∼2° in the 5° and 7.5° masked conditions, respectively.

## Discussion

The results show that some voxels in the cortical representations of the central visual field also respond when more peripheral locations are stimulated alone. These responses give rise to larger and more eccentric pRF estimates. Standard 2.5×2.5×2.5 mm^3^ fMRI voxels capture of the order of 10^6^ neurons [11], [12] that will have a variety of receptive field properties, and the pRF estimate only captures their central tendency. This central tendency measure will necessarily change if only a biased subset of the neural population is activated. This scenario would occur if the masked stimuli primarily activated neurons with large or eccentric receptive fields. Under such circumstances, the pRF estimates would register only the larger and more eccentric values of the subset of neurons activated by the peripheral stimulation (Figure 1). Similar arguments have been put forward in a review on adult V1 plasticity to explain receptive field changes following retinal lesions [13]. However, the key question is whether some of the neural receptive fields and the position scatter are indeed sufficiently large to be driven by stimulation beyond 5° or 7.5° eccentricity. From Figure 1 it seems that a rather large amount of RF variation would be required to cover the position shifts that we report, and the primate classical receptive field at low eccentricities does not extent sufficiently far into the periphery to cover the observed expansions [14]. The model presented in Figure 1 may therefore not be telling the full story.

**Figure 10:**
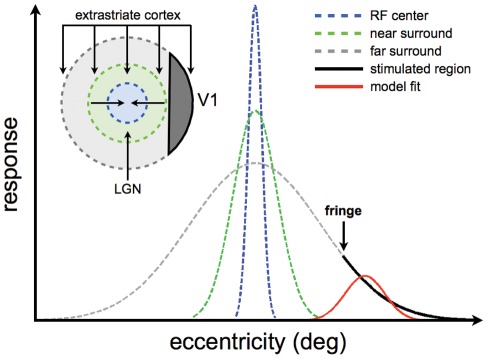
An explanation of the pRF dynamics in terms of feedback signals from extrastriate cortex. V1 neurons are presumed to have a receptive field (RF) center (dashed blue) that is measured by presenting high-contrast stimuli and commensurate with feed-forward connections from the lateral geniculate nucleus (LGN), a near surround (dashed green) that is measured by presenting low-contrast stimuli and commensurate with intra-areal V1 horizontal connections, and a far surround (dashed gray) that is commensurate with extrastriate feed-back connections [18], [20], [21]. We further assume that the pRF, which is measured by presenting high-contrast checkerboard stimuli, is an estimate of the RF center (dashed blue) when there is no mask. Normally, the response to stimulating the RF center is modulated by suppressive feed-back signals from the far periphery of the visual field [20]–[23]. However, when there is little or no stimulus contrast on the receptive field center, these feedback signals can also be excitatory [18], [20], [21], [24]. Under such circumstances, the far surround will be partially stimulated, which results in the skewed response indicated by the thick black curve. Fitting a Gaussian receptive field model to these responses will necessarily be shifted towards the fringe of the mask. It will also be larger than the RF center if the far surround extents sufficiently far into the periphery. Interestingly, this model also predicts that increasing the size of the mask (shifting the fringe to the right) results in a decrease of the pRF expansion. This is indeed what appears to happen when the mask size increases from 5.0° to 7.5° (see Figure 4B and corresponding text).

Other work has also revealed BOLD signals in the central representations of the visual field as a result of peripheral visual stimulation in normally sighted individuals [15] and patients [16]. In these studies, the patterns of activity were dependent on task/stimulus combinations, and the results were therefore interpreted in terms of feedback signaling from higher order visual areas. Indeed, the wide-spread BOLD signals that give rise to the larger and more eccentric pRF estimates are consistent with the distant BOLD modulation found in the macaque [17], and may well reflect the anatomical substrate of the very long spatial interactions in single V1 neurons [18] and of human contrast perception [19]. These very long-range spatial interactions are thought to arise from very rapidly conducting feed-forward-feedback loops [20]–[23] between V1 and higher order visual regions. Therefore, it could be that feed-back signals from the far periphery, which facilitate the neuronal response at low levels of excitation [18], [20], [21], [24], are visible as a change in the BOLD signal when the center of the visual field is masked. Figure 10 illustrates the effect of these interactions for a single neuron in V1. While the models in Figures 1 and 10 both give rise to displaced and enlarged pRF estimates when the neuronal population is partially stimulated, it appears that only the model in Figure 10 can explain the extent of the pRF displacements and expansions.

Regardless of explanation, the results indicate broad spatial tuning at the population level. This may underlie perceptual phenomena such as color, brightness, texture, and motion filling-in [25]–[31]. When the pRF expands or shifts away from its original to a more eccentric visual field location, the neuronal population will erroneously signal the presence of central stimuli to afferent neuronal populations [32], [33]. A similar mechanism might also account for the Delboeuf illusion [34] or perceptual distortions seen in patients with damage to the afferent visual pathways following stroke [35]. It could be interesting, therefore, to examine whether the pRF changes are also present under conditions in which these illusions and perceptual distortions occur. Furthermore, it has been observed that the receptive fields that were initially displaced and enlarged due to retinal lesions in animals, subsequently reduce in size towards the completion of the reorganization process (e.g., [36]). In adult human patients with macular lesions, only pRF expansions and displacements can be seen [8]. The most parsimonious explanation for these two observations is that the enlarged and displaced receptive fields provide a basis for long-term structural changes, but that these long-term structural changes do not necessarily follow through in human adulthood. To test this hypothesis, it would be worth studying the pRF characteristics in individuals with congenital loss of foveal vision, who do appear to exhibit cortical reorganization in the form of remapping [37].
